# Pelvic Osteosarcoma: Outcomes of Surgically Treated Patients in a Retrospective Single-Center Study

**DOI:** 10.3390/cancers18050738

**Published:** 2026-02-25

**Authors:** Tymoteusz Budny, Jan Christoph Theil, Georg Gosheger, Nils Deventer, Marieke de Vaal, Anna Maria Rachbauer, Niklas Deventer

**Affiliations:** 1 Department of Orthopedics and Tumororthopedics, University Hospital Münster, Albert-Schweitzer-Campus 1, 48149 Muenster, Germany; tymoteusz.budny@ukmuenster.de (T.B.); c.theil@orthopaedie-prinzipalmarkt.de (J.C.T.); georg.gosheger@ukmuenster.de (G.G.); mariekemathilda.devaal@ukmuenster.de (M.d.V.); anna.rachbauer@ukmuenster.de (A.M.R.); 2 Department of General Paediatrics, University Children’s Hospital Muenster, Albert-Schweitzer-Campus, 48149 Muenster, Germany; nils.deventer@ukmuenster.de

**Keywords:** pelvic osteosarcoma, oncology, musculoskeletal tumors, survival, prognostic factors, functional outcome

## Abstract

(1) Background: Pelvic osteosarcoma accounts for a small proportion of osteosarcoma cases but is associated with significantly poorer outcomes than extremity tumors. This study evaluates contemporary survival outcomes and prognostic factors in a single-center cohort. (2) Methods: We retrospectively analyzed 56 patients with primary pelvic osteosarcoma treated between 2006 and 2019. Demographic characteristics, surgical margins, adjuvant therapies, local recurrence, metastasis, survival outcomes and the Musculoskeletal Tumor Society (MSTS) Score were assessed. Kaplan–Meier analysis was performed for overall survival (OS), including subgroup analyses by age and Enneking classification. (3) Results: Median age at surgery was 24 years. R0 margins were achieved in 96.4% of cases. OS at 1, 3, and 5 years was 69%, 54%, and 48%, respectively. Younger patients (≤25 years) showed significantly improved 5-year OS (68%) compared with older groups. Enneking classification showed limited prognostic discrimination. Metastatic disease at any time strongly predicted inferior survival (5-year OS 30% vs. 66%). The mean MSTS score one year after operation was 14.1 points. Functional outcome showed marked variability and was strongly influenced by patient age, extent of resection, reconstruction strategy, and postoperative complications. Younger patients and those undergoing limited or non-acetabular reconstructions achieved superior functional results, whereas complex endoprosthetic reconstructions and revision-requiring complications were associated with reduced MSTS scores. (4) Conclusions: Pelvic osteosarcoma continues to be associated with substantial morbidity and mortality. Younger age and absence of metastatic disease are strong predictors of improved survival. Functional outcomes are typically moderate; further advances are needed to improve results.

## Simple Summary

Pelvic osteosarcoma is a rare but highly challenging malignancy due to the complex anatomy of the pelvis and the difficulty of achieving wide surgical margins. In this retrospective single-center study of 56 patients, we evaluated survival outcomes and prognostic factors including age, metastatic status, Enneking classification, histological subtype, response to neoadjuvant chemotherapy, and the functional outcome. Overall survival reached 48% at 5 years, with younger patients demonstrating significantly better outcomes. Achieving R0 resection was common and likely contributed to improved results compared to historical cohorts. Metastatic disease remained the strongest predictor of poor survival. The mean MSTS score one year after operation was 14.1 points. These findings provide updated insight into the contemporary management and prognosis of pelvic osteosarcoma and highlight the persistent challenges associated with this aggressive tumor.

## 1. Introduction

Pelvic osteosarcoma is one of the most challenging malignant bone tumors to treat. Although osteosarcoma is the most common primary bone sarcoma, pelvic involvement accounts for only 7–9% of all cases and is consistently associated with poorer outcomes than extremity disease [1,2,3]. Multiple interconnected factors contribute to this discrepancy: complex regional anatomy, late presentation, large tumor volumes, frequent neurovascular involvement, and difficulties in obtaining adequate surgical margins. The pelvis represents one of the most surgically challenging skeletal regions, limiting the feasibility of wide resections comparable to those achievable in limb osteosarcoma.

Historically, survival outcomes for pelvic osteosarcoma have lagged behind those of extremity tumors. Whereas modern cooperative group studies report 5-year overall survival (OS) rates of 65–75% in extremity osteosarcoma, pelvic osteosarcoma rarely exceeds 40%, with several landmark cohorts reporting 5-year OS between 20% and 30% [1,2,3]. Local recurrence rates exceeding 50% were common in earlier series, underscoring the technical difficulty of obtaining negative margins.

Over the past two decades, incremental improvements have emerged. Advances in preoperative imaging, computer-assisted navigation, custom three-dimensional implants, optimized chemotherapy regimens, improved patient selection, and concentration of care in high-volume sarcoma centers have contributed to better early and mid-term outcomes [2,3,4]. Nevertheless, pelvic osteosarcoma remains a dominant contributor to osteosarcoma-related mortality.

The Enneking classification has been applied widely to musculoskeletal tumors, although its prognostic value in pelvic osteosarcoma remains inconsistent. More contemporary anatomic classifications—including iliosacral-based systems—attempt to capture the complexity of pelvic resections more accurately, but their prognostic relevance remains debated [4,5].

The objective of this study was threefold: (1) to characterize clinical outcomes in a contemporary single-center cohort of pelvic osteosarcoma; (2) to identify prognostic variables including age, metastatic status, and anatomical classification; and (3) to compare these findings with major historical cohorts.

## 2. Materials and Methods

### 2.1. Study Design

A retrospective cohort study was performed including all patients with histologically confirmed pelvic osteosarcoma treated at our tertiary referral center between 2006 and 2019. Ethical approval was given by the local ethics committee (Ethics committee of the Westphalia-Lippe Medical Association and the Westphalian Wilhelms University of Münster, Germany; reference number 2020-317 F-s). Patients were identified via the hospital information system. Inclusion criteria were: (1) primary osteosarcoma of pelvic origin; (2) surgical treatment with curative intent (patients deemed unresectable were not part of this cohort); and (3) minimum follow-up of three months unless death occurred earlier. Exclusion criteria included secondary malignancies, recurrent disease prior to treatment at our institution, and incomplete medical records.

### 2.2. Clinical Variables

Data were extracted regarding patient demographics, age at surgery, sex, tumor grading, histological subtype, Enneking classification, chemotherapy (neoadjuvant/adjuvant), radiotherapy, metastatic disease at diagnosis or during follow-up, local recurrence, type of hemipelvectomy, reconstruction method, surgical margins, postoperative complications, and survival.

### 2.3. Imaging and Diagnostic Workup

All patients underwent standardized staging consisting of MRI of the pelvis, CT of the thorax, and PET-CT or bone scintigraphy. Tumors were staged according to the Enneking staging principles; however, due to inconsistent documentation, anatomical descriptors sometimes replaced conventional staging categories.

### 2.4. Surgical Treatment

Surgical resections were classified based on operative reports and pathological assessments. All surgical specimens were assessed by specialized musculoskeletal pathologists according to institutional standardized protocols. Resection margins were defined histologically as wide (R0), marginal (R1), or intralesional (R2). No marginal resections were reclassified post hoc.

**Figure 1:**
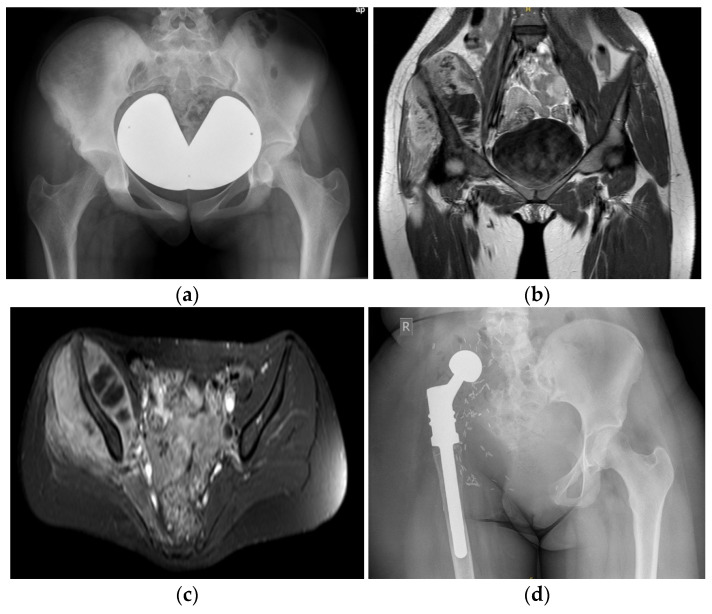
(**a**–**d**) Preoperative X-ray (**a**) of a 15-year-old male patient with an extensive high-grade osteosarcoma of the right ilium; preoperative MRI scans ((**b**), coronal view; (**c**), axial view); postoperative X-ray (**d**) after P1-4 hemipelvectomy and reconstruction with a modular endoprosthesis and attachment tube.

In cases involving the acetabulum, hip transposition was performed, securing the femoral head to the remaining sacrum using an attachment tube (Trevira; Implantcast, Buxtehude, Germany) and suture anchors (Mitek Super Anchor; DePuy Synthes, Raynham, MA, USA). For isolated iliosacral resections, iliolumbar reconstruction was carried out using pedicle screws, spinal rods, and polymethylmethacrylate. In patients (Figure 1) with involvement of the hip joint or proximal femur, an extra-articular resection was undertaken, and the proximal femur was reconstructed using a modular endoprosthesis (modular tumour and revision system (MUTARS); Implantcast, Buxtehude, Germany). Navigation-assisted osteotomies and three-dimensional preoperative planning tools were implemented in complex resections, particularly during the later years of the study period.

### 2.5. Chemotherapy and Radiotherapy

Neoadjuvant chemotherapy typically consisted of cisplatin, doxorubicin, and high-dose methotrexate. Histologic response to neoadjuvant chemotherapy was assessed using the Saltzer-Kuntschik score. Adjuvant chemotherapy was administered depending on postoperative recovery and histological response. Radiotherapy was administered selectively in cases of local recurrence or metastatic disease. All patients received photon-based external beam radiotherapy. Doses ranged from 54 to 66 Gy for local recurrence or 37–45 Gy for metastatic lesions. Radiotherapy was delivered postoperatively.

### 2.6. Functional Outcome

Functional outcome was assessed using the Musculoskeletal Tumor Society (MSTS) score for the lower extremity. The MSTS instrument evaluates six functional domains (pain, function, emotional acceptance, use of supports, walking ability, and gait), each rated from 0 to 5, resulting in a total score ranging from 0 to 30.

MSTS scores were extracted retrospectively from follow-up documentation one year postoperatively. Patients without an available MSTS score were excluded from MSTS-specific analyses but remained in the oncologic survival analyses.

### 2.7. Statistical Analysis

Survival analyses were performed using the Kaplan–Meier method. Patients were censored at the date of last documented follow-up if no event had occurred. Given the median follow-up duration of 11.5 months, particular caution was applied when interpreting long-term survival estimates beyond 24 months.

To evaluate independent prognostic factors, univariable and multivariable Cox proportional hazards regression models were performed. Variables entered into the multivariable model included age group, metastatic status, Enneking Classification, histological response and margin status. Hazard ratios (HR) with 95% confidence intervals (CIs) were calculated. A *p*-value < 0.05 was considered statistically significant.

The data analysis software used was Python Version: 3.4. (Python Foundation, Wilmington, DE, USA).

## 3. Results

### 3.1. Patient Characteristics

A total of 56 patients with primary pelvic osteosarcoma were included in the analysis. The median age of the patients at surgery was 24 years (range 2–72 years), with 29 patients (51.8%) aged ≤25 years, 14 patients (25.0%) aged 26–50 years, and 13 patients (23.2%) older than 50 years. Thirty-seven patients (66%) were male and 19 (34%) female. The median follow-up time was 11.5 months (range 0–137). Tumor grading was available for 54 patients, with all patients presenting with high-grade G3 lesions (n = 54; 96.4%). Two patients (3.6%) had missing grading data.

Metastatic disease occurred in 25 patients (44.6%) at any time during the disease course, whereas 31 patients (55.4%) remained metastasis-free.

### 3.2. Treatment Modalities

Most patients received neoadjuvant chemotherapy (53/56; 94.6%) and a slightly smaller proportion underwent adjuvant chemotherapy (49/56; 87.5%). Radiation therapy was administered in seven cases (12.5%), typically in the setting of local recurrence or metastatic disease.

Histopathological findings were available for 56 patients. Remarkably, R0 resection margins were achieved in 54 patients (96.4%). One case was classified as having missing or indeterminate margins, one case was classified as marginal resection. In multivariable Cox regression analysis margin status could not be reliably estimated due to the extremely high R0 resection rate (96.4%) and lack of variation, resulting in model instability.

### 3.3. Overall Survival (OS)

With a median follow-up time consistent with the survival dataset, the 1-year, 3-year, and 5-year OS rates were 68.7%, 54.2%, and 47.9%, respectively. The median overall survival was 41 months (Table 1).

**Figure 2:**
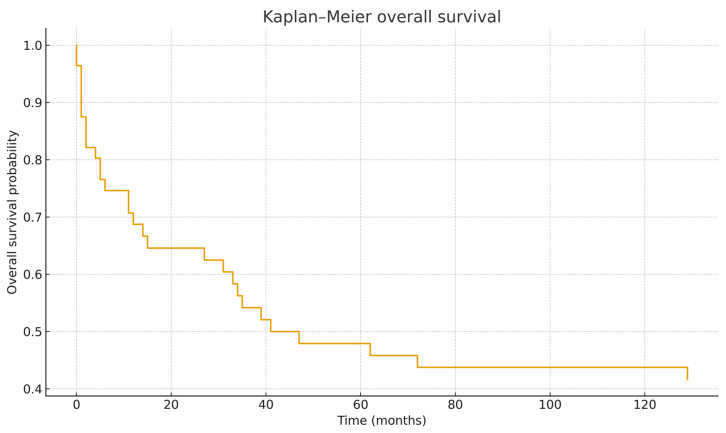
Kaplan–Meier Overall Survival.

The Kaplan–Meier curve (Figure 2) demonstrates the steepest decline during the first 24–36 months.

### 3.4. Recurrence-Free Survival (RFS)

Due to a combination of extensive censoring and lower event frequency in early follow-up, the calculated 1-year, 3-year, and 5-year RFS rates were all approximately 100%, and the median RFS was not reached. Given the relatively short median follow-up duration and the degree of right-censoring, long-term survival estimates beyond two years should be interpreted with caution. The apparent plateau in recurrence-free survival likely reflects censoring structure rather than true absence of recurrence events.

### 3.5. Histological Subtype

Histological subtype was available in 54 of 56 patients (96.4%). The majority of tumors were classified as conventional osteoblastic osteosarcoma (n = 39; 69.6%). Conventional chondroblastic osteosarcoma accounted for 10 cases (17.9%), while conventional poorly differentiated fibroblastic osteosarcoma was observed in 4 patients (7.1%). One patient (1.8%) was diagnosed with telangiectatic osteosarcoma. In two cases (3.6%), histologic subtype was not clearly documented (Table 2). The TNM stage was only indicated in individual cases in the pathological reports (n = 8/56).

Given the predominance of conventional osteoblastic subtype, subgroup survival comparisons were performed using simplified grouping (osteoblastic vs. non-osteoblastic), but no statistically significant survival differences were observed (HR 0.840, 95% CI 0.34–2.02, *p* = 0.690) (Table 2).

### 3.6. Histologic Response

Histologic response to neoadjuvant chemotherapy according to the Saltzer-Kuntschik score was available in 50 patients (89%). Thirteen patients (26%) were classified as good responders (grade 1–3), whereas 37 patients (74%) were poor responders (grade 4–6). Mortality rates were comparable between groups (53.8% vs. 51.4%). Median follow-up was longer among good responders (25 months) compared with poor responders (11 months). In this cohort, histologic response did not demonstrate a clear association with overall survival; however, interpretation is limited by sample size and follow-up heterogeneity (good response (SK < 3) HR 1.28, 95% CI 0.52–3.15, *p* = 0.592).

### 3.7. Survival by Age Group

Subgroup analysis demonstrated a clear survival advantage for younger patients. At 5 years, OS rates were:

≤25 years: 68.2%;

26–50 years: 35.7%;

>50 years: 30.8%.

**Figure 3:**
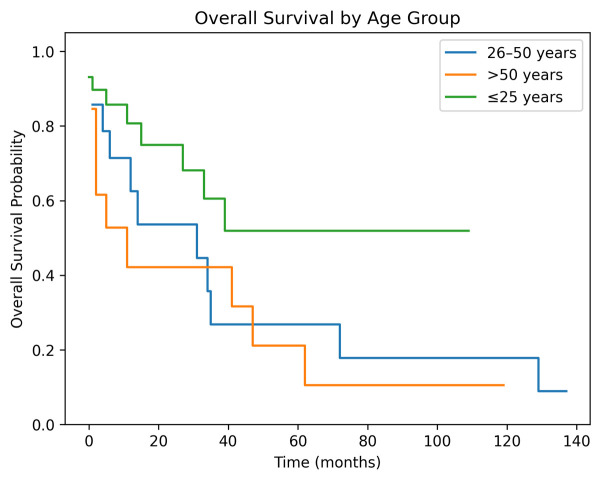
Overall Survival by Age Group.

The survival curves (Figure 3) show early separation between groups, with younger patients demonstrating a more favorable trajectory throughout the entire follow-up period.

A multivariable Cox proportional hazards model including age at surgery was performed. Increasing age showed a non-significant trend toward worse survival (HR 1.02 per year, 95% CI 1.00–1.05, *p* = 0.021) (Table 3).

**Table 3:** Multivariable Cox Regression (overall survival).

Variable	HR	95% CI	*p*-Value
Age at surgery (years)	1.02	1.00–1.05	0.021
Metastatic at diagnosis (yes)	2.63	0.98–7.10	0.056
Good response (SK ≤ 3)	1.28	0.52–3.15	0.592
Non-osteoblastic histology (vs. osteoblastic)	0.84	0.34–2.02	0.690

### 3.8. Survival by Enneking Classification

The Enneking classification demonstrated variable but overlapping OS distributions. Five main groups were present:

I, II, III, IV (n = 31): 5-year OS ≈ 49.3%;

I, II, IV (n = 7): 57.1%;

II, III (n = 7): 42.9%;

I, II, III (n = 4): 50.0%;

I (n = 4): 50.0%;

I, II (n = 1): 0% (single case).

Simplified grouping results in acetabulum + sacrum (n = 38), acetabulum only (n = 16) and no acetabulum/sacrum (n = 2) (Table 4).

**Table 4:** Simplified Enneking grouping.

Simplified Enneking Grouping	n	Deaths	KM 5-Year OS (%)
Acetabulum + sacrum	38	16	52.4
Acetabulum only	16	8	35.7
No acetabulum/sacrum	2	0	10.0

Although some groups displayed numerically higher survival, overlapping confidence intervals and limited sample size within subgroups limited statistical interpretation.

### 3.9. Influence of Metastatic Disease

Metastatic status was a strong predictor of survival. Patients without metastases had a 5-year OS of 66.4%, whereas those with metastatic disease had a significantly poorer 5-year OS of 30.0%. A multivariable Cox proportional hazards model including metastatic disease was performed. Metastatic disease was independently associated with inferior overall survival (HR 2.63, 95% CI 0.98–7.10, *p* = 0.056) (Table 3).

### 3.10. Multivariable Cox Regression Analysis

In multivariable Cox regression analysis (Table 3) including metastatic status at diagnosis, age group, resection margin (R0 vs. R1/R2), and Saltzer–Kuntschik histologic response, metastatic disease at diagnosis remained the only independent predictor of overall survival. Patients presenting with metastases demonstrated a significantly increased hazard of death compared with non-metastatic patients. Age, margin status, and histologic response did not retain independent statistical significance after adjustment. The absence of a margin effect should be interpreted cautiously given the very high proportion of R0 resections in the cohort, which limited statistical variability.

### 3.11. Functional Outcome

Functional outcome was assessed using the Musculoskeletal Tumor Society (MSTS) score for the lower extremity, which was available for 36 of 56 patients (64.3%) at one year follow-up assessment. The mean MSTS score for the entire evaluated cohort was 14.1 ± 4.2 points, corresponding to 47% of the maximum achievable score.

Age at surgery was associated with functional outcome. Younger patients achieved higher MSTS scores, with a gradual decline observed with increasing age: ≤25 years: 15.1 ± 4.2 (n = 21), 26–50 years: 13.0 ± 3.2 (n = 9), >50 years: 12.3 ± 5.3 (n = 6).

Metastatic status did not significantly influence functional outcome. Patients without metastases achieved a mean MSTS score of 13.8 ± 4.5 (n = 21), while patients with metastatic disease had a mean score of 14.5 ± 3.9 (n = 15).

Functional outcome varied across reconstruction strategies. Patients without reconstruction or with biologically limited reconstructions showed slightly higher MSTS scores, whereas complex reconstructions were associated with lower average functional results. Mean MSTS scores according to reconstruction type were: no reconstruction: 15.0 ± 4.8 (n = 4), hip transposition: 14.0 ± 5.1 (n = 12), modular endoprosthetic reconstruction: 13.8 ± 3.8 (n = 16), other reconstruction techniques: 15.0 ± 3.7 (n = 4). Overall, no reconstruction method demonstrated clear functional superiority, underscoring that functional outcome is influenced by multiple factors beyond implant choice alone.

Patients without postoperative complications demonstrated a numerically higher functional outcome compared with those who experienced at least one complication. The mean MSTS score was 14.7 ± 3.9 in patients without complications (n = 18) versus 13.5 ± 4.6 in patients with complications (n = 18). Although functional impairment was more pronounced in the complication group, the difference did not reach statistical significance.

### 3.12. Complications

Postoperative complications occurred in 32 of 56 patients (57.1%). At least one surgical revision or wound-related complication was required in the majority of affected patients.

## 4. Discussion

Pelvic osteosarcoma remains one of the most technically challenging and prognostically unfavorable subtypes within malignant bone tumors. The present analysis contributes recent institutional results in a well-defined cohort and allows comparison with updated long-term survival data. Historically, pelvic osteosarcoma cohorts demonstrated 5-year survival rates between 18% and 34%, with outcomes largely limited by unresectable disease, intralesional margins, and early metastatic progression [1,2,3,4]. Against this background, our 5-year overall survival (OS) of nearly 48% appears encouraging and reflects gradual improvements in staging, careful patient selection, perioperative optimization, and institutional experience. These trends parallel broader shifts toward earlier referral, standardized treatment algorithms, and centralization of pelvic tumor surgery to specialized centers [5,6,7].

A key message reinforced by this study concerns the important prognostic effect of metastasis at diagnosis. Patients who presented with metastatic disease showed clearly inferior OS and shorter follow-up durations, confirming findings from large registry-based analyses and multicenter cohorts [8,9,10]. Metastatic presentation often coincides with high tumor burden, biologically aggressive disease, and reduced resectability. Importantly, several authors have demonstrated that this negative effect persists despite intensified systemic treatment, supporting the concept that metastatic disease marks an advanced biological phenotype rather than merely delayed detection [9,10,11]. Our data align with these observations: patients without metastasis achieved an OS exceeding 66%, approaching survival levels reported for extremity osteosarcoma.

Age likewise emerged as a consistent prognostic factor. Younger patients experienced better survival, likely reflecting superior chemotherapy tolerance, fewer comorbidities, and greater feasibility of functionally demanding resections. Prior pediatric and AYA-focused cohorts reported similar age-dependent gradients [6,8,12]. Conversely, older patients often face limitations related to frailty and treatment intolerance. Our data suggest that the age effect persists even after accounting for margin status, indicating interaction between host biology and treatment feasibility rather than age alone.

Margin status remains one of the few clinically modifiable parameters in pelvic osteosarcoma. Historically, intralesional resections were common due to proximity to major vessels, pelvic viscera, and sacral neural structures. Earlier series documented local recurrence rates exceeding 60–70% when margins were intralesional or marginal [1,2,3,4]. In contrast, our cohort achieved an exceptionally high rate of R0 resections. This likely reflects careful patient selection, precise planning, multidisciplinary coordination, and institutional expertise. Nevertheless, the exceptionally high R0 rate observed in this cohort warrants careful interpretation: only patients undergoing surgery with curative intent were included. Patients deemed unresectable were not part of this cohort. All resections were performed in a high-volume tertiary referral center with standardized pathological assessment protocols and frequent use of advanced preoperative planning techniques. While this likely contributed to improved margin quality, referral bias inherent to specialized centers cannot be fully excluded. Recent literature consistently emphasizes that margin quality—rather than reconstruction type—drives local control and survival [13,14,15]. Adjuncts such as navigation-assisted osteotomies and patient-specific cutting guides may further reduce unexpected positive margins, although they remain supportive tools rather than replacements for profound oncologic judgement [14,16].

The distribution of histological subtypes in our cohort reflects the known predominance of conventional osteoblastic osteosarcoma, which typically accounts for approximately 60–75% of pelvic cases in large series [1,2,3]. Chondroblastic and fibroblastic variants are less frequent, while telangiectatic osteosarcoma remains rare. Previous studies [1,2,3,4] have not consistently demonstrated a strong independent prognostic impact of histological subtype in pelvic osteosarcoma. In our cohort, no clear survival difference was observed between osteoblastic and non-osteoblastic tumors. However, the limited number of non-osteoblastic cases restricts definitive conclusions.

Although histologic response to chemotherapy represents a well-established prognostic factor in extremity osteosarcoma, our cohort did not demonstrate a clear survival difference between good and poor responders. This may reflect the dominant impact of surgical resectability and metastatic status in pelvic disease, as well as limited statistical power.

Tumor size continues to function as both a biological and technical surrogate. Larger tumors are frequently associated with metastatic presentation, inferior margins, and decreased survival. Large multicenter cohorts have consistently demonstrated significantly worse OS for tumors ≥ 10 cm [4,8,11,17]. Notably, survival becomes comparable between pelvic and extremity osteosarcoma once tumor volume is matched, suggesting that pelvic tumors are not inherently more aggressive but are often diagnosed later [7,17]. These findings highlight the importance of timely referral and early imaging for persistent pelvic pain or atypical neurologic symptoms.

Sacral involvement adds considerable complexity. Extension into or across the sacral ala limits true en-bloc resection, increases neurologic morbidity, and elevates the risk of local failure despite nominally negative margins [5,13,18]. Multicenter analyses have shown higher recurrence in sacral extension, emphasizing the anatomical limits of radicality. In selected cases, integration of advanced radiotherapy techniques may offer meaningful local control when radical resection is not feasible [19,20,21].

Local recurrence remains one of the most decisive clinical events in pelvic osteosarcoma. Recurrence is frequently followed by systemic dissemination and poor salvage rates. Our cohort demonstrated comparatively low recurrence frequency, likely attributable to consistent R0 resections, rigorous preoperative planning and careful patient selection. Nevertheless, vigilant surveillance remains essential because delayed detection markedly worsens prognosis [1,2,3,4].

Radiotherapy has re-emerged as an important adjunct. Historically used primarily for palliation, proton and carbon-ion therapy now allow precise, conformal dose escalation while limiting exposure to adjacent organs. Several contemporary studies reported promising local control in non-, borderline-resectable or margin-positive disease [20,21,22]. However, long-term toxicity data remain incomplete, and radiotherapy should complement rather than replace surgery whenever feasible.

Postoperative morbidity is substantial in pelvic osteosarcoma surgery. In our cohort, complications occurred in more than half of patients, consistent with previous reports [12,23,24]. Deep infection, wound complications, and mechanical failure remain common. Importantly, complications did not appear to independently compromise oncologic survival, reinforcing that margin achievement remains the dominant determinant while complications primarily affect functional recovery and quality of life.

Reconstruction techniques continue to evolve. Traditional saddle prostheses and early modular devices were associated with high failure and infection rates [23,24]. More contemporary patient-specific implants and hybrid reconstructions aim to improve biomechanical fit and may reduce mechanical complications [15,16]. Although these innovations enhance function, they have not consistently altered oncologic survival, underscoring that biological clearance remains essential.

Functional outcome after pelvic osteosarcoma resection remains a major concern and represents a key patient-centered endpoint beyond oncologic survival. In the present cohort, the mean MSTS score of 14.1 points corresponds to approximately 47% of normal lower extremity function, reflecting the substantial functional impairment associated with extensive pelvic resections.

Several previous studies have reported comparable functional outcomes following internal hemipelvectomy. Wirbel et al. observed mean MSTS scores in the moderate range after pelvic tumor reconstruction and identified acetabular resection as a key determinant of inferior function [18]. Similarly, Abudu et al. demonstrated that pelvic endoprosthetic reconstructions, particularly those involving the periacetabular region, are associated with substantial functional compromise despite acceptable oncologic control [25]. These findings align closely with our results and support the notion that restoration of near-normal lower limb function after pelvic osteosarcoma surgery remains uncommon.

The relationship between postoperative complications and functional outcomes warrants particular attention. In our cohort, patients who experienced major postoperative complications tended to demonstrate lower MSTS scores, reflecting prolonged immobilization, delayed rehabilitation, and the cumulative burden of revision procedures. Comparable associations have been reported previously, with deep infection, wound breakdown, and mechanical failure consistently linked to inferior functional recovery [7,12]. Importantly, while complications may not independently compromise oncologic survival, they significantly affect quality of life, mobility, and long-term independence, underscoring the importance of complication prevention and early multidisciplinary management.

Reconstruction strategy also plays a decisive role in functional outcome. Patients requiring complex reconstructions, especially following acetabular resection, generally demonstrated lower MSTS scores compared with those undergoing more limited iliac or iliosacral resections without hip joint involvement. This observation mirrors findings from multiple series reporting inferior functional performance after Enneking type II pelvic resections, regardless of reconstruction method [12,25]. Although advances in patient-specific implants and three-dimensional reconstruction techniques have improved implant fit and stability, their impact on functional outcomes appears limited, suggesting that loss of native hip biomechanics and muscular attachments remains the dominant constraint [23,25].

Age and systemic disease status further influence postoperative function. Younger patients in our cohort tended to achieve higher MSTS scores, likely reflecting superior rehabilitation potential, fewer comorbidities, and greater tolerance of aggressive surgery. Conversely, patients with metastatic disease often demonstrated reduced functional outcomes, which may be attributable to cumulative treatment burden, systemic therapy-related toxicity, and earlier disease progression. Similar trends have been reported in other sarcoma populations, emphasizing that functional recovery is closely linked to both host factors and overall disease progression [9,11].

Beyond objective functional scores, quality of life after pelvic osteosarcoma surgery is shaped by chronic pain, gait impairment, reliance on walking aids, and reduced participation in social and professional activities. Previous studies have shown that even patients with acceptable MSTS scores frequently report persistent limitations in daily living and reduced health-related quality of life [22]. These observations highlight that functional scores alone may underestimate the long-term impact of pelvic resections and reinforce the need for comprehensive survivorship care, including physiotherapy, pain management, and psychosocial support.

When contextualized within the broader literature, our oncological outcomes appear comparable to—or slightly improved over—those reported in many historical cohorts [1,3,6,26]. However, comparisons across the literature are challenging due to heterogeneous study designs. Gradual improvements likely reflect treatment centralization, more refined systemic therapies, and advances in surgical planning [3,5,7,24].

This study has several limitations that should be acknowledged. First, its retrospective and single-center design introduces inherent risks of selection bias, incomplete data capture, and heterogeneity in documentation across the study period. Treatment strategies, imaging technology, and surgical techniques evolved substantially over time and cannot be fully controlled for. Second, although this represents one of the larger institutional cohorts of pelvic osteosarcoma, the overall sample size remains limited, and subgroup analyses may be underpowered. Third, the relatively short median follow-up duration represents an important methodological limitation. Although 5-year survival estimates are reported for comparability with historical cohorts, censoring structure significantly influences long-term Kaplan–Meier projections. The high recurrence-free survival estimates at later time points should therefore not be interpreted as definitive disease control but rather as a function of limited follow-up and event distribution. Functional outcome data were not available in standardized form but extracted from routine clinical documentation. No formal interobserver reliability testing was performed, and follow-up intervals were not prospectively standardized. Furthermore, one-year functional outcomes may not fully capture long-term adaptation after complex pelvic reconstruction. These limitations necessitate cautious interpretation of functional findings. Chemotherapy and radiotherapy were not protocolized, introducing treatment variability.

Despite these limitations, the strengths of this study include a well-defined cohort, uniform histologic confirmation, detailed surgical documentation, and long-term follow-up. These data provide clinically relevant insight into contemporary pelvic osteosarcoma management.

## 5. Conclusions

In summary, pelvic osteosarcoma remains a formidable oncologic challenge. However, our results indicate that with margin-oriented surgery performed in specialized centers, with integrated multimodal treatment, and with thoughtful patient selection, long-term outcomes can be meaningfully improved. Functional outcomes are typically moderate and strongly influenced by acetabular involvement, reconstruction complexity, and postoperative complications.

## Figures and Tables

**Table 1:** Survival Outcomes (OS).

Outcome	Estimate
1-year OS	68.7%
3-year OS	54.2%
5-year OS	47.9%
Median OS	41 months

**Table 2:** Histological Subtype.

Histological Subtype	n	Percentage (%)
Conventional osteoblastic	39	69.6
Conventional chondroblastic	10	17.9
Conventional fibroblastic (poorly differentiated)	4	7.1
Telangiectatic	1	1.8
Missing	2	3.6

## Data Availability

The data presented in this study are available on request from the corresponding author. The data are not publicly available due to legal, ethical and privacy issues.
